# Decoding the Methylome: Insights into the Epigenetic Regulation of Polycystic Ovarian Syndrome through Mitochondrial DNA Methylation

**DOI:** 10.2174/0113892029401274251009070350

**Published:** 2025-10-22

**Authors:** Aparna Eledath Kolasseri, Ramasamy Tamizhselvi, Sivaraman Jayanthi

**Affiliations:** 1 School of Biosciences and Technology, Vellore Institute of Technology, Vellore 632014, Tamil Nadu, India

**Keywords:** PCOS, epigenetics, mitochondrial DNA methylation, bisulfite sequencing, machine learning, artificial intelligence

## Abstract

Polycystic Ovarian Syndrome (PCOS) imposes significant societal health and economic challenges. The precise determinants behind the global prevalence of PCOS are still poorly understood. However, epigenetic modifications in PCOS, such as DNA methylation, have been recognized as a method by which the environment interacts with the genome. Evidence suggests that changes in mitochondrial (mt)DNA methylation may have a role in the heightened occurrence of PCOS. This article provides a comprehensive overview of nuclear DNA methylation, mitochondrial DNA methylation, and their significance in regulating gene expression. Pre-existing scholarly works shed insight into the complex interaction of DNA methylation and other epigenetic modifications associated with PCOS. In addition, this review gathers a detailed explanation of several methodologies employed to assess alterations in DNA methylation at specific sites and across the nuclear and mitochondrial genomes. Integrating mtDNA methylation alterations may be a promising diagnostic strategy for PCOS, potentially paving the way for novel therapeutic interventions.

## 1 INTRODUCTION

Polycystic Ovary Syndrome (PCOS) is a hormonal disorder that impacts the health of females during their reproductive age. It is linked to various health conditions, including hypertension, type 2 diabetes mellitus (T2DM), insulin resistance (IR), dyslipidemia, hyperandrogenemia, endometrial cancer, and psychiatric disorders [1]. The prevalence of PCOS is estimated to be between 5.5 and 12.6% in women aged from 17 to 45 years worldwide. In India, the prevalence estimate for the same group of people ranges from 8.2% to 22.5% based on the Rotterdam diagnostic criteria. The complexity of this condition stems from its typical heterogeneity and the unknown aetiology [2].

PCOS is one of the leading causes of infertility in females. Being obese and overweight, having a sedentary lifestyle, and having a family history of PCOS may predispose a young female to PCOS. The risk of developing metabolic disorders and glucose intolerance in PCOS patients eventually increases by 11-fold compared with a same-aged control group. Pharmaceutical treatments are crucial for managing PCOS. Combined oral contraceptives help regulate menstrual cycles and lessen the symptoms of hyperandrogenism. Many PCOS-afflicted women use Metformin to improve insulin sensitivity and ovulation. Compared to clomiphene, letrozole has shown higher fertility success rates. Emerging therapies with promise include GLP-1 receptor agonists, antiandrogens, and inositol supplements [3, 4]. Along with these medications, proper diagnosis and lifestyle changes involving a balanced, healthy diet and weight loss may help alleviate the long-term adverse effects of PCOS. Although the cause of PCOS is unknown, studies point to environmental and lifestyle variables, epigenetic effects, endocrine disturbances, and genetic susceptibility as predisposing factors [5, 6].

Defective granulosa cells (GC) cause impaired follicular development, dysregulated insulin signaling, and aberrant ovarian steroidogenesis, particularly in theca cells, resulting in excessive androgen production and altered cellular communication within the ovary, often accompanied by genetic predisposition and influenced by factors such as inflammation and obesity [7, 8]. The pathophysiology of PCOS is mostly characterized by hormonal abnormalities, which include insulin resistance, increased androgens, and disturbed gonadotropin production. In addition to causing irregular menstruation, hyperandrogenism also plays a role in the hirsutism and acne that are hallmarks of PCOS [9]. Follicle cysts develop as a result of ovarian function being disrupted by dysregulated gonadotropins. Insulin resistance creates a feedback loop by increasing testosterone production. Recent research highlights the complex hormonal interactions and the critical role that hormonal dysregulation plays in the onset and expression of PCOS [6, 10].

Numerous genes and pathways mediating PCOS have been identified. Genes for androgen biosynthetic pathways, like Cytochrome P450(CYP) family genes like CYP11a, CYP21, CYP17, and CYP19, are responsible for the synthesis of steroids in the body and have controversial properties in the occurrence of PCOS. Besides, the androgen receptor (AR) gene, sex hormone binding globulin gene (SHBG), leutinizing hormone receptor (LH), follistatin gene, follicle-stimulating hormone receptors (FSHR), insulin gene (INS), and insulin receptors (INSR) are among the most investigated gene pathways in PCOS [11, 12]. Accumulating evidence suggests that the androgen gene located on the X chromosome, acting through AR, is a crucial factor in the onset of PCOS [5, 13]. Numerous studies on heritable epigenetic modifications, such as miRNA, DNA methylation, and histone acetylation, which play a role in the occurrence and development of the condition, may herald a possible target for the therapy of PCOS [14, 15]. Notably, CAG repeats and DNA methylation patterns in the AR may alter signal transduction, thereby contributing to the development of PCOS [16, 17].

The current review highlights the underlying mechanisms and efficacy of regulating nuclear and mitochondrial DNA methylation in diagnosing and treating PCOS, particularly concerning ovarian function and overall female health. Additionally, it encompasses the vast majority of current procedures that have been proven effective in detecting Mitochondrial DNA methylation.

## 2 METHODOLOGY

This mini-review was conducted by searching peer-reviewed literature across databases, including Scopus, PubMed, Web of Science, and Google Scholar. Keywords such as “*Polycystic ovarian syndrome”, “Mitochondrial dysfunction”, “Epigenetics”, “DNA methylation”, “Mitochondrial DNA methylation*”, “*Techniques for DNA methylation analysis”,* and “*Machine learning in PCOS diagnosis”* were used individually and in combination.

Articles published between 2010 and 2024 were considered. Selection criteria included original research articles and high-impact and review papers relevant to the pathophysiology, epigenetics, and techniques for identifying the methylation status in the PCOS condition. Additionally, we focused on machine learning approaches, as they represent a rapidly advancing area in the diagnosis of PCOS. Preference was given to articles published in English and indexed in high-impact journals. The inclusion of articles was determined by their quality, relevance, and contribution to the state of knowledge. To draw attention to new ideas and knowledge gaps, important findings were extracted and thematically synthesized.

## 3 GENETIC DYNAMICS: NAVIGATING EPIGENETIC REGULATION IN PCOS

Epigenetics is often considered a fundamental factor that regulates the basic genetic phenotype of PCOS. Studies have shown that epigenetic modifications induced by unfavourable environments in utero or after birth may cause PCOS-like symptoms in the adolescent group. Therefore, epigenetic changes that occur in ovaries, adipose tissue, skeletal muscle, and brain tissue are possibly found in females who all suffer from PCOS [17]. When ovarian tissues are challenged by critical components of the neural-immune-endocrine axis, immune cells quickly modify the transcription of many genes associated with PCOS manifestation. Rising evidence suggests that the regulation of PCOS progression is based on epigenetic reprogramming by altering DNA methylation, histone acetylation, and noncoding RNA-associated chromatin remodeling [18]. Specifically, the DNA methylation-mediated epigenetic regulations are widely discussed in PCOS women.

### 3.1 DNA Methylation and PCOS

Extensive evidence demonstrates the interaction of DNA methylation and other epigenetic processes in PCOS. Understanding the effects of various regulatory layers, notably DNA methylation, histone acetylation, and microRNAs (miRNAs), requires effective coordination among them. This section discusses the interconnected roles and potential therapeutic applications of these factors in PCOS.

A previous study revealed that 5-methylcytosine (5mc) and 5-hydroxymethyl cytosine (5hmc) signatures are substantially regulated by DNA methylating and demethylating enzymes, such as DNA methyltransferases (DNMT1, DNMT3A, DNMT3B) [19] and ten-eleven translocation (TET 1, TET2, TET3), respectively, in PCOS [20]. These enzymes regulate gene expression patterns associated with metabolic and reproductive disorders. The regulator of mitochondrial biogenesis, Peroxisome proliferator-activated receptor gamma coactivator 1-alpha (PPARGC1A) expression is decreased, which is connected to elevated blood androgen levels, insulin resistance, and reduced mitochondrial DNA(mtDNA) content [21-23]. Moreover, the PPARGC1A promoter's hypermethylation contributes to the emergence of PCOS by causing insulin resistance, metabolic dysfunction, and abnormalities in reproduction, thus indicating the significance of epigenetic dysregulation in the pathogenesis of the condition [21].

The luteinizing hormone choriogonadotropin (LHCG) gene promoter exhibits lower DNA methylation levels in PCOS, and its overexpression leads to the production of more LH by GC, which in turn causes gonadotropin dysfunction in PCOS [24]. According to research conducted by Mao *et al.,* sudden DNA methylation affects the expression of genes related to cellular processes, such as lipid and steroid synthesis, as well as carbohydrate metabolism, which contribute to PCOS pathogenesis [25]. Recent research has disclosed that increased production of androgens can contribute to ovarian dysfunction by altering epigenetic changes, particularly DNA methylation, thereby contributing to the occurrence and development of PCOS [26]. Liu *et al.* discovered that the degree of methylation of the nuclear receptor corepressor 1 (NCOR1) promoter in GCs was decreased in patients with PCOS hyperandrogenism compared to patients with PCOS without hyperandrogenism and non-PCOS patients [27]. Similarly, the occurrence of DNA methylation was elevated in the promoter region of the peroxisome proliferator-activated receptor gamma (PPARG1), leading to a decrease in the production of genes that promote PPARG1. PPARG1 regulates ovarian function, whereas NCOR1 acts as a nuclear corepressor of PPARG1 that is essential in hormonal signalling associated with reproductive functions [28-30]. Through methylation analysis in GC of PCOS patients, it is revealed that CpG hypomethylation of genes like mastermind-like domain containing 1 (MAMLD1), growth hormone-releasing hormone receptor (GHRHR), aldo-keto reductase family 1 member C3 (AKR1C3), and resistin, as well as hypermethylation of tumour necrosis factor-alpha (TNF), can indirectly cause androgen excess [8]. Subsequently, hormonal imbalance may contribute to DNA methylation and the genes involved in PCOS. However, the understanding of the effects of androgens on the regulation of DNA methylation patterns under PCOS conditions is still in the exploration stage.

Gene expression and epigenetic changes can be influenced by insulin signalling. Patients with PCOS-IR have metabolic dysfunction, including IR, while those without IR have hormonal and reproductive issues but no significant metabolic impairment [31]. The methylation of the CCAAT/enhancer-binding protein (C/EBP) gene was found to differ significantly between PCOS-IR patients and PCOS non-IR patients when the regulatory network of differentially expressed genes was examined [32]. Reduced methylation of the gene epoxide hydrolase 1 enzyme suppresses testosterone and increases the risk of PCOS [33]. Also, higher circulating testosterone is correlated with increased DNA methylation in genes involved in the cell cycle process [27]. Amyloid beta precursor protein and parkin gene expression could potentially be recognized from cord blood samples of PCOS women and could have diagnostic implications, pointing to intrauterine reprogramming and epigenetic markers that raise the risk for PCOS for the growing fetus [34].

Specifically, the mutual interplay loop between DNA methylation and miRNA expression in PCOS was recently substantiated by strong evidence. Transcriptional alterations in metabolic diseases and genome-wide DNA methylation were found in GCs, ovaries, and adipose tissue of PCOS patients [35]. Specifically, the molecules targeted by altered miRNA expression were found in follicular fluid, GCs, serum, peripheral blood leukocytes, and adipose tissue, all associated with PCOS [36]. The CpG islands in the promoter regions of miRNAs can become hypermethylated as a result of DNA methylation, which inhibits the miRNA transcriptional activation. When the promoter of a miRNA-coding gene is hypermethylated, it results in miRNA silencing events; conversely, when a miRNA promoter is hypomethylated, it leads to miRNA overexpression [37, 38]. The expression of their corresponding genes, such as X-linked inhibitor of apoptosis (XIAP), bromodomain-containing protein-3 (BRD3), mitogen-activated protein kinase-14 (MAPK14), and solute carrier family 7 number 5 (SLC7A5), is also found to be regulated by DNA methylation of the miR-429, miR-141-3p, and miR-126-3p promoter regions, respectively [26]. These miRNAs were suppressed by promoter hypermethylation, resulting in decreased expression in women with PCOS [39]. These findings suggest that the promoters of miRNAs are targets for CpG island methylation, leading to an epigenetic interplay between miRNA and DNA methylation associated with PCOS.

The PCOS-induced depression is influenced by elevated acetylcholine levels and neuroinflammation, interlinked with increased DNA methylation mediated by DNMT and HDAC2 in the hippocampus. HDAC2 physically interacts with DNMT1, DNMT3A, and DNMT3B, forming repressive complexes that couple histone deacetylation with DNA methylation [40]. Moreover, the reduced PPARG expression in GCs is associated with DNA hypermethylation mediated by increased DNMT1 activity. It is correlated with increased PPARG expression in human adipose tissue mediated by histone 3 lysine 4 (H3K4) methylation [41]. Further, differential occupancy of H3K9 acetylation with reduced H3K9 methylation is linked with hypomethylation of CpG sites in promoters of CYP19A1 in cumulus cells in PCOS [42]. The experimental PCOS model showed a significant reduction of DNA methylation and dimethylation of H3K9 in oocytes, which was correlated with increased acetylation of H4K12. Additionally, mRNA expression of DNMT1 and HDAC1 was significantly decreased in the PCOS-like mice model, with a significant correlation between increased production of reactive oxygen species and excessive histone acetylation, which is a novel finding and may provide new insights into the mechanism causing PCOS [43].

Altogether, the PCOS epigenetic machinery exhibits a strong correlation between DNA methylation and histone deacetylation, and there is also a connection with neuronal control that disrupts the epigenomic landscape, contributing to the development of PCOS.

### 3.2 Unlocking the Link: Mitochondrial Dysfunction and DNA Methylation in PCOS

Growing evidence suggests that epigenetic mechanisms may regulate mtDNA in PCOS. Patients with PCOS have persistently high levels of inflammatory markers due to low-grade, chronic inflammation. Recent studies have generated interest in mitochondrial dysfunction, suggesting that oxidative stress may promote the production of proinflammatory cytokines in PCOS. Oxidative stress, brought by an imbalance between antioxidants and reactive oxygen species (ROS), contributes to PCOS and its complications. Excessive ROS production leads to mitochondrial dysfunction and attenuates hypoxia-inducible factor-1/Bcl-2 interacting protein-3 mediated mitophagy in the ovaries of PCOS rats [44]. These findings suggest that proper mitochondrial functioning regulates the progression of PCOS.

Excessive production of ROS promotes the pro-inflammatory gene expression that disrupts the blood and follicular fluid of the ovaries and oocyte development, which eventually causes chronic inflammation in PCOS patients. Also, women with PCOS have increased levels of inflammatory markers such as TNF-α, CRP, IL-6, IL-8, IL-18, free radical formation, and malondialdehyde levels, along with lower total antioxidant capacity (TAC) [45]. Also, targeted metabolomics revealed alterations in intermediate metabolites from glycolysis, TCA cycle, branched-chain amino acid catabolism, fatty acid oxidation, and NAD catabolism in follicular fluid from classic PCOS patients. Additionally, cumulus cells from PCOS patients showed signs of increased oxidative stress, mitochondrial malfunction, and unbalanced redox potential. Oocyte development and follicle growth abnormalities in PCOS women are significantly affected by mitochondrial activity and intermediate metabolism in follicular fluid [46].

The integrity and copy number of mtDNA are crucial for sustaining cellular energy homeostasis. A decline in mtDNA copy number is linked to mitochondrial dysfunction, resulting in lower ATP production, heightened oxidative stress, and impaired insulin signaling associated with metabolic disorders [34]. Women with PCOS are more prone to develop metabolic disorders that show dramatic changes in peroxisome proliferator-activated-receptor gamma-coactivator 1 alpha (PGC-1α) promoter methylation and mtDNA copy number [47]. PGC-1α, a key regulator of mitochondrial biogenesis, which, when altered, can lead to impaired energy metabolism and contribute to conditions such as obesity, type 2 diabetes, and cardiovascular disorders [48-50]. Mutations in another gene involved in mitochondrial biogenesis, mitochondrial transcription factor A (TFAM), were associated with a higher risk of PCOS in the Indian population. Similarly, the lower level of TFAM and associated PGC-1α was found in South Indian women with PCOS [51]. A study conducted on Chinese women with PCOS showed that elevated mitochondrial copy number acts as an indicator for the early stages of PCOS. Also, certain mitochondrial D-loop mutations and haplotypes seem to offer protection against PCOS [52]. In contrast, Lee *et.al* found that the number of mitochondrial copies was decreased in the peripheral blood of women with PCOS condition [53]. There is a strong correlation between the level of serum testosterone and mitochondrial copy number, which shows a time-dependent decrease in patients treated with metformin over one year [54]. These findings show that mitochondrial biogenesis, copy number changes, and D-loop mutations could be possible biomarkers for PCOS conditions.

Methylation of mtDNA occurs at CpG dinucleotides; however, due to its smaller size and fewer noncoding regulatory regions, mtDNA contains fewer CpG islands. In humans and higher-order animals, the methylation of adenine and cytosine at the C-5 position results in N6-methyladenine (6mA) and 5-methylcytosine (5mC), respectively, both of which play a crucial role in gene regulation. DNMTs generated in the nucleus are converted into mitochondrial DNA methyltransferase (mtDNMTs) in the mitochondria. Adenine is methylated by methyltransferase, like N-4 and N-6 adenine-specific DNA methyltransferase 1. In PCOS, mtDNA methylation in response to oxidative stress may be directly linked to mitochondrial dysfunction, which is associated with greater inflammatory markers and poorer antioxidant capacitance [19]. Researchers observed that DNMT expression was associated with decreased mitochondrial Sirtuin 3 expression, which regulates mitochondrial proteins *via* acetylation in GCs of PCOS women [55].

Mitochondrial metabolism regulates the synthesis of the universal methyl donor (SAM), which is essential for both nuclear and mitochondrial DNA methylation. Co-substrates of histone phosphorylation and acetylation, as well as deacetylation, are generated in the mitochondria. Therefore, these organelles are associated with all histone modifications and DNA methylation processes in a specific cell [56]. Dysfunction of mitochondria and the low quality of oocytes produced in polycystic gilt ovaries are linked to aberrant one-carbon metabolism activation and hypermethylation of mtDNA. Homocysteine (Hcy) content in the follicular fluid was noticeably higher in polycystic ovaries. The highly activated one-carbon metabolic pathway upregulated DNMT1 expression, and mtDNA hypermethylation all occurred at the same time as the increased Hcy in the follicular fluid. These metabolic and epigenetic alterations may explain the altered mitochondrial function and poor oocyte quality in PCOS gilt ovaries [46]. The summarized findings in Table **1** highlight the genes affected by aberrant epigenetic marks in PCOS, classified according to their cellular location—nuclear, mitochondrial, or nuclear with mitochondrial functions and their involvement in key biological processes associated with the PCOS condition.

These epigenetic alterations may act independently or may lead to other modifications, ultimately regulating the gene expression associated with PCOS. The role of mitochondrial epigenetics (methylation) and its complications is an emerging area of research in PCOS, and it may serve as a promising diagnostic and therapeutic target for the prevention or treatment of PCOS. Fig. (**1**) depicts the above-mentioned underlying mechanisms in nuclear and mitochondrial methylation that play a major role in PCOS pathogenesis.

## 4 TECHNIQUES IN FOCUS: SHEDDING LIGHT ON MITOCHONDRIAL DNA METHYLATION IN PCOS PATHOGENESIS

Several methods are available for determining the DNA methylation status in the emerging field of epigenetics. Nevertheless, selecting the best and appropriate method remains a challenging endeavor. The chosen method should provide an unbiased response to the query being posed by the researcher.

Here, the available methods for identifying differential methylation in the nuclear genome, which can also be applied to the identification of methylation in the mitochondrial genome in PCOS conditions, are described.

### 4.1 Isolation of the Mitochondrial Genome for the Methylation Detection

An organism’s entire genome is made up of both nuclear and mtDNA. To avoid introducing potential nuclear mitochondrial pseudogenes (NUMTs) contamination, the mitochondria must first be isolated [57]. Some effective techniques for isolating mt DNA from tissues include (A) using Percoll gradients, (B) using linear DNA digestion, (C) using differential centrifugation, (D) using a commercial kit for rapid differential centrifugation, and (E) using magnetic separation of mitochondria with anti-TOM22 antibodies [58]. After isolating the mitochondrial genome, specific techniques for DNA methylation analysis are listed in Table **2**.

### 4.2 Artificial Intelligence and Machine Learning: The Approach to Revolutionizing PCOS- Epigenetics Research

Machine learning (ML) is a domain of artificial intelligence that utilizes massive datasets to predict future events. It has the potential to help physicians make accurate diagnoses in the medical field, as well as in genetics and epigenetics, as part of personalized medicine [74]. Machine learning can accurately diagnose PCOS using FSH and LH indicators [75].

ML methods can discover epimutation-prone genomic areas, improving illness diagnosis. Epigenomic research using ML-based bioinformatics and high-throughput data has made major advancements. Machine learning predicts epigenomic data mostly *via* imbalanced class learning, active learning, and deep learning [76]. Using a large outpatient population, the researchers effectively built ML algorithms to predict PCOS, suggesting the potential for early detection and intervention. Hormonal levels and obesity were significant positive predictors of PCOS, while gravidity and positive bHCG were negative predictors, underlining critical aspects that can help in diagnosis [77]. Tiwari *et al.* diagnosed PCOS with a machine learning-based smart polycystic ovarian syndrome diagnostic system (SPOSDS). A variety of machine-learning PCOS screening methods are evaluated using non-invasive screening parameters [78]. Metabolomics and ML models can be used to diagnose PCOS by examining steroid profiles. The modelling process categorizes steroid contributions to support the biomarker approach and reveal pathophysiological pathways [79]. Machine learning techniques in the epigenetics field can be applied to predicting epigenetic sites, forecasting biological parameters, diagnosing and prognosing diseases, and enhancing and advancing epigenetic analysis. Artificial Intelligence (AI), particularly deep learning, is being applied to epigenomic research to accelerate the discovery and development of new treatments [80]. Combining traditional and innovative analytical strategies can be used to identify etiopathogenic epimutations that are disease-specific, such as those employed in microarray and pyrosequencing [81]. EWASplus is a computational ML-based method that extends Epigenome-Wide Association Studies (EWASs) coverage to the entire genome and predicts hundreds of new significant CpG sites [82].

**Fig. (2):**
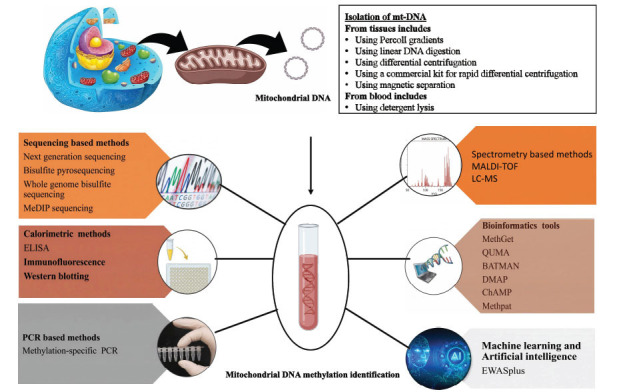
Analysis of Mitochondrial and Nuclear DNA methylation in PCOS: Different methods for accurate identification of DNA methylation in mitochondrial and nuclear DNA of PCOS patients.

Many elements of mtDNA methylation remain unexplained. Global and gene-specific variations in mtDNA methylation patterns may be linked to diseases or risk factors associated with PCOS, although more research is needed. The use of machine learning strategies is not specifically focused on PCOS research. Therefore, the above-explained techniques could be useful for individuals seeking to learn more about mtDNA methylation analysis in PCOS. Fig. **2** depicts the diagrammatic representation of the methods mentioned.

## CONCLUSION

In conclusion, mitochondrial epigenetics is a relatively new field of study, with increasing evidence suggesting that mtDNA methylation and other epigenetic changes play a significant role in the development of PCOS. These abnormal methylation patterns are increasingly associated with mitochondrial dysfunction, which leads to hormonal imbalance and impaired metabolic regulation. New methods are being developed for early diagnosis and intervention when these mitochondrial epigenetic alterations are identified as potential biomarkers or therapeutic targets. Furthermore, our capacity to recognize and decipher these molecular signatures has improved due to recent developments in high-throughput technologies, machine learning, and artificial intelligence. This review highlights the combined roles of nuclear and mitochondrial epigenetic mechanisms in PCOS pathophysiology, with a particular focus on mitochondrial DNA methylation as a promising yet underexplored area for the development of future diagnostic tools and targeted therapies.

## Figures and Tables

**Fig. (1):**
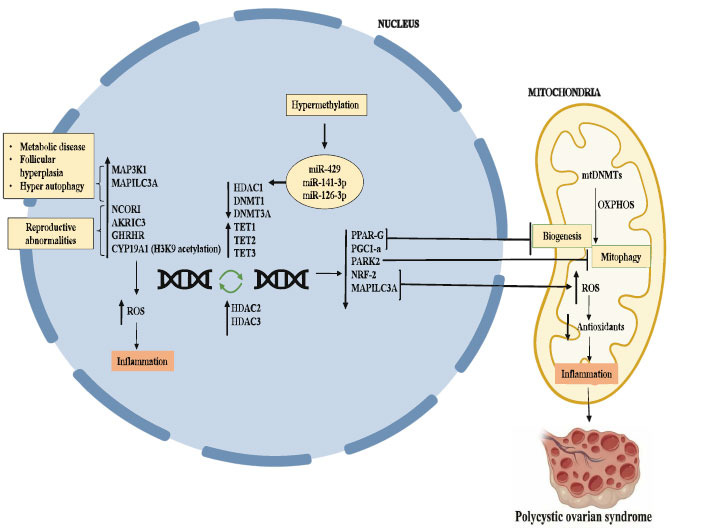
Mitochondrial DNA methylation in PCOS pathogenesis: Nuclear and Mitochondrial dysfunction caused by DNA methylation is a potential trigger for metabolic diseases, reproductive abnormalities, and inflammation, and finally leads to PCOS.

**Table 1:** The genes affected by abnormal epigenetic marks in PCOS with cellular and functional classification.

**Gene**	**Epigenetic Alteration**	**Category**	**Role in PCOS**
PPARGC1A (PGC-1α)	Promoter hypermethylation	Nuclear with mitochondrial function	Regulates mitochondrial biogenesis; altered methylation leads to metabolic dysfunction.
PPARG1	Promoter hypermethylation	Nuclear	Regulates ovarian and adipose tissue functions; downregulation is linked to IR and hormonal imbalance.
TET1, TET2, TET3	DNA demethylation enzymes (convert 5mC to 5hmC); dysregulated	Nuclear	Alter the methylation landscape in PCOS by promoting DNA demethylation
NCOR1	Promoter hypomethylation	Nuclear	Nuclear corepressor of PPARG; affects reproductive hormone signaling
LHCG	Promoter hypomethylation	Nuclear	Overexpression causes a gonadotropin imbalance
MAMLD1	CpG hypomethylation	Nuclear	Associated with androgen excess and steroidogenesis
GHRHR	CpG hypomethylation	Nuclear	Growth hormone signaling; linked to hyperandrogenism
AKR1C3	CpG hypomethylation	Nuclear	Involved in steroid metabolism and androgen production
Resistin	CpG hypomethylation	Nuclear	Adipokine involved in insulin resistance
TNF	Promoter hypermethylation	Nuclear	Inflammatory cytokines contributing to PCOS pathogenesis
C/EBP	Altered promoter methylation, histone acetylation	Nuclear	Regulates adipogenesis, steroidogenesis, and inflammation;
XIAP	Downregulated *via* miRNA promoter methylation	Nuclear	Inhibitor of apoptosis; suppressed *via* miR-429 methylation
BRD3	Downregulated *via* miRNA promoter methylation	Nuclear	Epigenetic reader protein; suppressed *via* miR-141-3p methylation
MAPK14	Downregulated *via* miRNA promoter methylation	Nuclear	Involved in stress response; suppressed *via* miR-126-3p methylation
SLC7A5	Downregulated *via* miRNA promoter methylation	Nuclear	Amino acid transporter; indirectly influenced by epigenetic miRNA regulation
CYP19A1 (Aromatase)	Promoter hypomethylation (H3K9 acetylation linked)	Nuclear	Key enzyme in estrogen synthesis; altered in cumulus cells of PCOS
Epoxide hydrolase 1	Promoter hypomethylation	Nuclear	Linked to testosterone levels and PCOS risk
Amyloid beta precursor protein	Hypomethylated (cord blood)	Nuclear	Suggested diagnostic marker linked to fetal programming
Parkin	Hypomethylated (cord blood)	Nuclear with mitochondrial function	Implicated in mitophagy and mitochondrial quality control
TFAM	Reduced expression; methylation-linked	Nuclear with mitochondrial function	Key regulator of mtDNA transcription and replication
SIRT3	Downregulated (linked to DNMT expression)	Nuclear with mitochondrial function	Regulates mitochondrial protein deacetylation
DNMT1, DNMT3A, DNMT3B	Upregulated	Nuclear	DNA methyltransferases modulate both nuclear and mitochondrial DNA methylation
HDAC1, HDAC2	Altered expression	Nuclear	Histone deacetylases form repressive complexes with DNMTs
mtDNA (various loci)	Hypermethylation (5mC, 6mA)	Mitochondrial	Linked to mitochondrial dysfunction and inflammatory responses in PCOS
Mitochondrial D-loop	Epigenetically altered/haplotypes	Mitochondrial	Biomarker potential; implicated in mitochondrial stability
SAM-related enzymes (one-carbon metabolism)	Upregulated	Mitochondrial	It drives DNA methylation, linked to homocysteine buildup and poor oocyte quality.

**Table 2:** Methods for DNA methylation analysis in the Nuclear and Mitochondrial genomes.

**Methods**	**Applications**	**References**
Sequencing-based methods		
Next-generation sequencing (NGS)	Providing a comprehensive and unbiased view of the epigenome	[59, 60]
Bisulfite pyrosequencing or Whole genome bisulfite sequencing (WGBS*)*	Detect individual CG methylation at various levels from PCR amplicons of a region up to 115bp in length	[61, 62]
Calorimetric methods		
MethylFlash	Methylated DNA Quantification Kit and using antibodies quantified by measuring absorbance at 450 nm in an ELISA-like reaction	[63, 64]
Immunofluorescence method	It allows robust, sensitive, and quantitative investigation of global DNA methylation patterns through immunofluorescence, flow cytometry, and fluorescence microscopy.	[65, 66]
Western blotting	Expression of HDAC1, HDAC2, HDAC3, and DNMT1 can be detected	[67, 68]
PCR-based methods for the detection of methylation		
Methylation Specific PCR (MSP)	To determine methylation of the D-loop region of mtDNA using bisulfite-modified DNA	[32, 69]
Spectrometry-based methods for DNA methylation identification		
Matrix-assisted laser desorption ionization-time of flight mass spectrometry (MALDI-TOF)	Permits high-throughput methylation site detection and semi-quantitative measurement at single or multiple CpG positions	[70, 71]
Liquid chromatography-mass spectrometry (LC-MS)	Uses DNA ion exchange columns to extract mtDNA from genomic DNA	[72, 73]

## LIST OF ABBREVIATIONS

5hmc: 5hydroxy Methyl Cytosine
5mc: 5methyl Cytosine
6mA: 6methyl Adenine
AGK: Acylglycerol Kinase
AI: Artificial Intelligence
AKR1C3: Aldo-keto Reductase Family 1 Member C3
APP: Amyloid beta Precursor Protein
AR: Androgen Receptor Gene
BME: Bagging Meta-estimator
BRD3: Bromodomain-containing Protein-3
CAT: Catalase
CEBP: CCAAT/enhancer-binding Protein
COBRA: Combined Bisulfite Restriction
CRP: C-reactive Protein
CYP11a: Cytochrome P450 Family Genes like 11a
CYP17: Cytochrome P450 Family 17
CYP19: Cytochrome P450 Family 19
CYP21: Cytochrome P450 Family 21
DHEA: Dehydroepiandrosterone
DMAP: Differential Methylation Analysis Package
DNMT: DNA Methyltransferases
DT: Decision Tree
E2: Estradiol
EDC: Endocrine-disrupting Chemicals
EPHX1: Epoxide Hydrolase1
EWAS: Epigenome-Wide Association Studies
FSHR: Follicle-stimulating Hormone Receptors
GB: Gradient Boosting
GC: Granulosa Cells
GHRHR: Growth Hormone Releasing Hormone Receptor
HAT: Histone Acetyl Transferase
Hcy: Homocysteine
HDAC: Histone Deacetylase
HIF-1/BNIP3: Hypoxia-inducible factor-1/Bcl-2 interacting protein-3
hMeDIP-seq: Hydroxy methylated DNA Immunoprecipitation along with Next-generation Sequencing
IL-6: Interleukin-6
IL-8: Interleukin-8
INS: Insulin Gene Insulin Receptors (INSR)
KNN: K Nearest Neighbour
LC-MS: Liquid Chromatography-mass Spectrometry
LH: Leutinizing Hormone
LR: Logistic Regression
MALDI-TOF: Matrix-assisted Laser Desorption Ionization-time of Flight Mass Spectrometry
MAMLD1: Mastermind-like Domain Containing 1
MAPK14: Mitogen-activated Protein Kinase-14
MCP-1: Monocyte Chemoattractant Protein-1
MDA: Malonaldehyde
METT: Methyltransferase like 4
ML: Machine Learning
MMP: Matrix Metalloproteinase
MPO: Myeloperoxidase
MSP: Methylation Specific PCR
mtDNA: mitochondrial DNA
mtDNMT: mitochondrial DNA Methyltransferase
mtFAS: Mitochondrial Fatty Acid Synthase
N6AMT1: N-6 Adenine-specific DNA Methyltransferase 1
NB: Naive Bayes
NCOR1: Nuclear Receptor Corepressor 1
nDNMT: nuclear DNA Methylatransferase
nDNMT3a: nuclear DNA Methylatransferase 3a
nDNMT3b: nuclear DNA Methylatransferase3b
nDNMTa: nuclear DNA Methylatransferase-a
NGS: Next-generation Sequencing
NLRP1: NLR Family pyrin Domain Containing 1
NUMT: Nuclear Mitochondrial Pseudogenes
OS: Oxidative Stress
OSI: Oxidative Stress Index
OXPHOS: Oxidative Phosphorylation
PARK2: Parkin
PCOS: Polycystic Ovarian Syndrome
PGC-1: Peroxisome Proliferator Activated-receptor Gamma-co Activator
QUMA: Quantification Tool for Methylation Analysis
RF: Random Forest
RF: Random Forest
SAM: S-Adenosyl Methionine
SERAC1: Serine Active Site Containing Protein-1
SHBG: Sex Hormone Binding Globulin Gene
SLC7A5: Solute Carrier Family 7 Number 5
SOD: Super Oxide Dismutase
SPODS: Smart Polycystic Ovarian Syndrome Diagnostic System
SVM: Support Vector Machine
T2DM: Type 2 Diabetes Mellitus
TAC: Total Antioxidant Capacity
TAOS: Total Antioxidant Status
TAZ: Tafazzin
TET: Ten-eleven Translocation
TFAM: Mitochondrial Transcription Factor A
TNF: Tumor Necrosis Factor
WBC: White Blood Count
WGBS: Whole Genome Bisulfite Sequencing
XGB: XG Boost
XIAP: X-linked Inhibitor of Apoptosis
XO: Xanthine Oxidase
